# One “misunderstood” health issue: demonstrating and communicating the safety of influenza a vaccination in pregnancy: a systematic review and meta-analysis

**DOI:** 10.1186/s12889-021-10740-w

**Published:** 2021-04-09

**Authors:** Qing Chun Lu, Tie Yun Zhang, Pravesh Kumar Bundhun, Cheng Chen

**Affiliations:** 1 Department of Obstetrics and Gynaecology, National Hospital of Guangxi Zhuang Autonomous Region, Affiliated to Guangxi Medical University, Nanning, Guangxi 530000 People’s Republic of China; 2 Department of Communication, School of Journalism and New Media, Xi’An JiaoTong University, Xi’An, Shanxi 710000 People’s Republic of China; 3 Department of Internal Medicine, Flacq Hospital, Central Flacq, Mauritius; 4 Department of Broadcasting and Television, School of Journalism and Information Communication, Huazhong University of Science and Technology, Wuhan, 430000 People’s Republic of China

**Keywords:** Health communication, Communication scholars, Influenza vaccine, Fetal outcomes, Pregnancy, Public health, Risk ratio, Centre of disease control and prevention

## Abstract

**Background:**

The American College of Obstetricians and Gynecologists (ACOG) makes certain recommendations including the annual influenza vaccination of pregnant and pre-pregnant women during influenza (flu) season with an inactivated influenza vaccine as soon as it becomes available. The Centers for Disease Control and Prevention’s (CDC) Advisory Committee on Immunization Practices in association with ACOG state that the vaccine is safe to be given any trimester during pregnancy. However, due to a lack of communication, the public is unaware of the effects of influenza A vaccination in pregnancy. Since this is a vital public health concern, we aimed to communicate with evidence, the safety of influenza A vaccination in pregnancy in order to improve the rate of influenza A vaccines in pregnant women.

**Methods:**

This health communication issue was based on the impact of influenza vaccine on fetal outcomes. Therefore, a search was carried out through medical-based online databases including: Cochrane Central, EMBASE, Web of Science, MEDLINE, http://www.ClinicalTrials.gov, and Google scholar for relevant English-based publications. Adverse fetal outcomes were considered as the endpoints of this analysis. The most specific RevMan 5.3 (latest version) software was used to carry out this analysis. Risk ratios (RR) with 95% confidence intervals (CI) were involved in data and results representation and interpretation.

**Results:**

A total number of 679, 992 pregnant women participated in this analysis. Based on this current analysis, premature/preterm birth (< 37 weeks) was significantly reduced in pregnant women who were vaccinated for influenza A (RR: 0.80, 95% CI: 0.69–0.92; *P* = 0.002) as compared to those women who were not vaccinated. Similarly, influenza A vaccination decreased the risk for very preterm birth (< 32 weeks) (RR: 0.70, 95% CI: 0.58–0.84; *P* = 0.0001). The risks for infants with low birth weight (RR: 0.71, 95% CI: 0.49–1.04; *P* = 0.08), very low birth weight (RR: 0.69, 95% CI: 0.23–2.11; *P* = 0.52) and infants small for gestational age (RR: 0.93, 95% CI: 0.83–1.05; *P* = 0.26) were not increased with the vaccine. Influenza A vaccination was not associated with increased risks of stillbirth (RR: 0.63, 95% CI: 0.38–1.03; *P* = 0.07), birth defects (RR: 0.67, 95% CI: 0.26–1.72; *P* = 0.41), admission to neonatal intensive care unit or Apgar score < 7 in 5 min.

**Conclusion:**

Influenza vaccine is completely safe in pregnancy. It significantly lowers premature birth and is not associated with any serious adverse neonatal outcome. Hence, this important piece of information should be communicated and conveyed to all pregnant women, for a safer and healthier pregnancy. At last, this public health issue should further be addressed to the population through media and other communication means in order to improve the rate of influenza A vaccines in pregnant women for a healthier and more productive population.

## Background

For a long time, “health” and “communication” have always been playing an important role in human production and life. As a concept, “health communication” was formally put forward in the 1970s. Similar to other concepts in Humanities and Social Sciences, people also have different opinions on health communication, forming multi-dimensional interpretations. However, the most classic definition is the view of Rogers, a pioneer in communication, “Health communication is any type of human communication whose content is concerned with health” [1]. Simply speaking, health communication is a kind of behavior that produces and delivers information related to human health. For example, a research work based on a specific health-related topic demonstrating and explaining certain guidelines and preventive measures could be shared among several groups of people who are concerned with the issue. In this process, communication plays the role of a “controller”.

Health communication has been introduced into public health and education for heath by American scholars since 1960s, enriching and developing the theory and methods of health education. In general, the Stanford Heart Disease Prevention Program (SHDPP) launched in the United States in 1971, is regarded as the real beginning of health communication research.

The ultimate purpose of health communication has been to make people form a change from cognition to action; to promote public and individual’s physical/mental health; to accomplish a harmonious operation in our whole society [2]. In other words, “health communication” has set up its mission to persuade the public of adopting health behaviors and preventive measures proven to be safe and effective. As such, Influenza A vaccination is an essential health concern during pregnancy.

Vaccination during pregnancy has often been a controversy among the current general population. People are not well informed, and prefer to avoid any kind of vaccination during pregnancy because they are unsure or unaware of its consequences or effects on the fetus [3]. Similarly, people are not aware of the importance of inactivated influenza vaccine during pregnancy [4]. Due to a lack of communication, the public is unaware of the benefit and they have a belief that, similar to other vaccines, influenza A vaccine is also contraindicated during pregnancy [5, 6].

In contrast, influenza vaccination is actually vital during pregnancy because influenza can result in severe health conditions including progression to pneumonia during antepartum and postpartum periods [7]. This might be associated with adverse perinatal and neonatal outcomes [8]. Hence, vaccination against influenza A during pregnancy might be protective.

The American College of Obstetricians and Gynecologists (ACOG) makes certain recommendations including the annual influenza vaccination of pregnant and pre-pregnant women during influenza (flu) season with an inactivated influenza vaccine as soon as it becomes available. The Centers for Disease Control and Prevention’s (CDC) Advisory Committee on Immunization Practices in association with ACOG state that the vaccine is safe to be given any trimester during pregnancy [9]. They also mention that maternal influenza immunization is an important constituent of maternal prenatal care as well as for the newborn. Medical Health Officers based in the department of Obstetrics and Gynecology, as well as other health care assistants are advised to counsel pregnant women about the safety and beneficial effects of influenza vaccination and passive immunity to their fetus.

Following the influenza A (H1N1) outbreak in the year 2009, even though influenza vaccine was first recommended to all pregnant women irrespective of gestational age in the United Kingdom in November 2010 [10], hesitancy was observed among this specific category of patients. There was a delay or a complete absence of acceptance of the vaccine despite its availability in vaccination services as stated by The Strategic Advisory Group of Experts on Immunization (SAGE) Working Group [11] and it was believed that this hesitancy was due to a lack of communications concerning the safety of influenza A vaccine and other challenging concerns during pregnancy rendering a total of only 45% of pregnant women based in the United Kingdom to take this influenza A vaccination [12].

Since this is a vital public health concern, we therefore aimed to demonstrate and communicate with evidence, the safety of influenza A vaccination in pregnancy in order to improve the rate of influenza A vaccines in pregnant women.

## Methods

### Search databases and search strategies

This health communication issue was based on the impact of influenza vaccine on fetal outcomes. Therefore, a search was carried out through medical-based online databases: Cochrane Central, EMBASE, Web of Science, MEDLINE, http://www.ClinicalTrials.gov, and Google scholar for English-based publications.

Searched indices which were used were restricted to:

“pregnancy and influenza vaccine”; “pregnancy and influenza”; “pregnancy and H1N1”; “pregnancy and vaccination”; “pregnancy, influenza and fetal outcomes”; “influenza vaccine and pregnancy outcomes”; “influenza and fetal outcomes”; “influenza A vaccine and pregnancy outcomes”; “H1N1 and pregnancy outcomes”; “influenza vaccination and pregnancy abnormalities”; “influenza A vaccination and pregnancy abnormalities”.

### Inclusion and exclusion criteria

Criteria for inclusion were based on studies which:

- Were randomized trials and observational studies (cohort, cross-sectional, retrospective, prospective studies);
- Compared pregnant women who were vaccinated versus those who were not vaccinated for influenza A (H1N1) and reported fetal outcomes as their clinical endpoints;
- Involved dichotomous data which could be used in this analysis; that is, data which used binary ‘success’ or ‘failure’ categories to describe the status of subjects.

Criteria for exclusion were based on studies which:

- Were systematic reviews, meta-analyses or literature reviews;
- Were case studies;
- Did not show the comparison of vaccinated versus unvaccinated pregnant women for influenza A (H1N1);
- Did not report fetal outcomes;
- Involved data which were irrelevant to this analysis;
- Were duplicated studies; that is, studies that repeated themselves in different search databases, and different studies that involved the same trial or observational cohort.

### Data extraction and quality assessment

Data were independently extracted from the original studies by three reviewers. Data which were extracted involved the surnames of the first author, the publication year of the original articles, the maternal and fetal outcomes, the type of study, the total number of pregnant women who were vaccinated versus those who were not vaccinated, the percentage of women who suffered from gestational diabetes mellitus, high blood pressure, with multiple pregnancies and those on folic acid supplementation, the type of influenza vaccine, the number of events in each category and the methodological quality of each study.

The methodological quality of the observational studies (cross sectional and cohort studies) were assessed based on the criteria of the Newcastle Ottawa Scale (NOS) [13] whereas the methodological quality of the randomized trials were assessed by the criteria suggested by the Cochrane Collaboration [14]. Grades were allotted (Grade A = low risk of bias, Grade B = moderate risk of bias, Grade C = high risk of bias).

Any disagreement was discussed and resolved by the corresponding author (Cheng Chen).

### Outcomes

Table 1 listed the fetal outcomes reported in the original studies.

**Table 1:** Outcomes reported and follow-up time period

Studies	Fetal outcomes
**Baum2015** [15]	Stillbirth, early neonatal death, preterm birth, very preterm, low birth weight, fetal growth restriction, full term, live birth
**Beau2014** [16]	Small for gestational age, neonatal pathology, preterm birth, pregnancy loss
**Chambers2013** [17]	Live birth, stillbirth, termination, congenital defects
**Chambers 2016** [18]	Major birth defects, small for gestation, spontaneous abortion, stillbirth, live birth, termination, preterm birth
**Cleary2014** [19]	Small for gestation, preterm, spontaneous birth < 37 weeks, admitted to neonatal unit, perinatal death, congenital anomaly, Apgar score < 3 at 1 min, Apgar score < 7 at 5 min
**Fabiani2015** [20]	Stillbirth, preterm birth < 37 weeks, very preterm birth < 32 weeks, low birth weight < 2500 g, very low birth weight < 1500 g, low 5 min Apgar score < 7, congenital malformation
**Fell2012** [21]	Preterm birth < 37 weeks, very preterm birth < 32 weeks, small for gestation, 5 min Apgar score < 7, fetal death
**Kallen2012** [22]	Stillbirth, preterm birth, low birth weight, small for gestation, congenital malformation, cardiac malformation, VSD/ASD, hypospadias, orofacial clefts, eye malformations
**Legge2014** [23]	Preterm birth < 37 weeks, low birth weight, small for gestational age
**Lin2012** [24]	Preterm delivery, low birth weight, ASD, stillbirth, hyperbilirubinemia neonatal, contact dermatitis, upper respiratory tract infection, respiratory distress
**Maas2015** [25]	Small for gestation, preterm < 37 weeks
**Olsen2016** [26]	Small for gestational age, preterm
**Pasternak2012** [27]	Major birth defects, preterm birth, low birth weight, small for gestational age
**Richards2013** [28]	Preterm birth (27–36 weeks), low birth weight, small for gestational age
**Rubinstein2013** [29]	Preterm birth < 37 weeks, low birth weight, very low birth weight, fetal mortality, Apgar score < 7 in 5 min, admission to neonatal ICU, fetal malformation
**Sheffield2012** [30]	Preterm < 37 weeks, major malformation, stillborn, neonatal ICU, neonatal death, neonatal pneumonia
**Steinhoff2012** [31]	Small for gestational age, low birth weight, preterm < 37 weeks
**Steinhoff2017** [32]	Preterm < 37 weeks, small for gestation
**Zerbo2017** [33]	Small for gestational age, preterm birth, low birth weight, admission to neonatal ICU, Apgar score < 7 within 1 and 5 min respectively

Abbreviations: *ICU* Intensive care unit, *VSD* ventricular septal defects, *ASD* Atrial septal defect

The fetal endpoints which were assessed in this meta-analysis were limited to the following:

- Preterm birth (< 37 weeks);
- Very preterm birth (< 32 weeks);
- Low birth weight (< 2500 g);
- Very low birth weight (< 1500 g);
- Small for gestational age;
- Stillbirth;
- Major birth defects;
- Admission to neonatal intensive care units (NICU);
- Apgar score < 7 in 5 min.

### Statistical analysis

The most specific RevMan 5.3 (latest version) software was used to carry out this analysis. Risk ratios (RR) with 95% confidence intervals (CI) were involved in data and results representation and interpretation.

A subgroup analysis of the outcomes with a *P* value less or equal to 0.05 was considered statistically significant for this study. Any P value above 0.05 was not statistically significant.

Heterogeneity in meta-analysis, which is also referred to as the variation in study outcomes between studies, was represented by the I^2^ statistic test which described the percentage of variation across the studies that was due to heterogeneity rather than chance. The larger the percentage of I^2^, the higher the heterogeneity.

A random effect statistical model was used for this analysis.

Sensitivity analysis [34] was also carried out by a leave one out analysis whereby one study was excluded at a time and a new analysis was carried out each time to observe if the results were influenced by any of the studies.

Publication bias was visually observed through plotted funnels.

### Ethical approval

Ethical or board review approval was not required for this systematic review and meta-analysis.

## Results

### Search outcomes

Following a thorough search from the online databases (PRISMA Guideline) [35], a total number of 963 publications were retrieved. Based on an initial assessment of the titles and abstracts, 798 publications were eliminated due to irrelevance.

One hundred and sixty five (165) full texts articles were then assessed based upon the inclusion and exclusion criteria.

Elimination of full texts articles were based on the following aspects:

- They were systematic reviews, meta-analyses and literature reviews (17);
- They were case studies or letters of correspondence (8);
- They reported only maternal outcomes but did not report fetal outcomes (17);
- They did not show comparison of vaccinated versus unvaccinated women (19);
- They consisted of irrelevant data (4);
- They were duplicated/repeated studies and data (82).

**Fig. 1:**
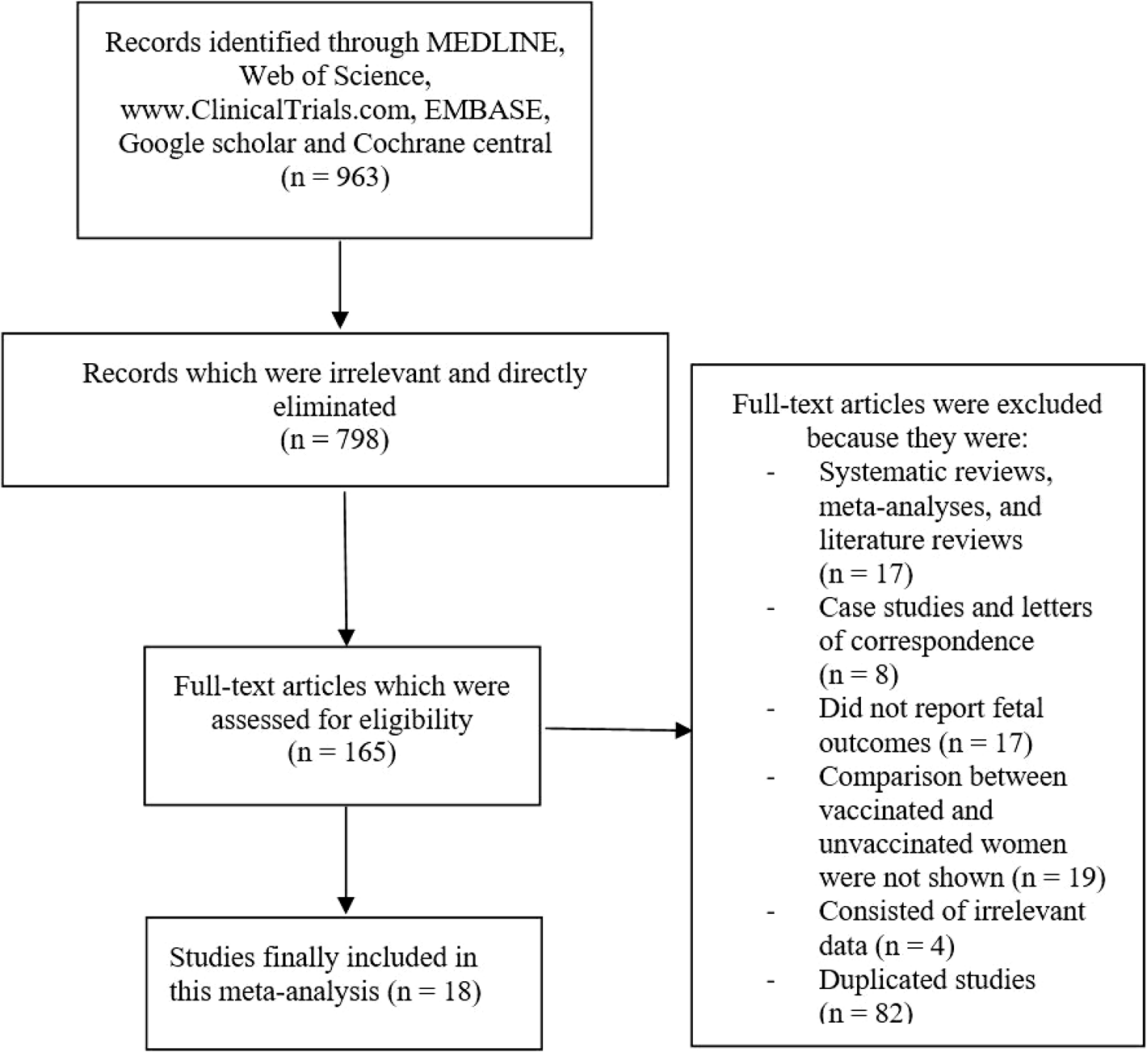
Flow diagram representing the study selection

Finally, 18 studies [15–33] were selected for this analysis as shown in Fig. 1.

### General and baseline features

A total number of 679, 992 pregnant women participated in this analysis. One hundred and eighty one thousand four hundred and seventy nine (181,479) pregnant women who were vaccinated were compared with 498,513 pregnant women who were not vaccinated for influenza A.

Table 2 lists the total number of participants which were extracted from each group. Two studies were randomized trials, 2 studies were cross sectional studies whereas the remaining 14 studies were cohort studies. The bias risk grade for each study was also listed in Table 2.

**Table 2:** General properties of the studies

Studies	No of women vaccinated (n)	No of women unvaccinated (n)	Type of study	Bias risk grade
**Baum2015** [15]	34,241	9363	Cohort study	B
**Beau2014** [16]	1645	3290	Cohort study	B
**Chambers2013** [17]	841	191	Cohort study	B
**Chambers 2016** [18]	1263	467	Cohort study	B
**Cleary2014** [19]	2996	3898	Cohort study	B
**Fabiani2015** [20]	2003	98,329	Cohort study	B
**Fell2012** [21]	23,340	32,230	Cohort study	B
**Kallen2012** [22]	18,612	83,298	Cohort study	B
**Legge2014** [23]	1958	10,265	Cohort study	B
**Lin2012** [24]	198	198	Cohort study	B
**Maas2015** [25]	1357	669	Cross sectional linkage study	B
**Olsen2016** [26]	2172	2931	Cohort study	B
**Pasternak2012** [27]	345 + 6644	22,917 + 46,443	Cohort study	B
**Richards2013** [28]	1125	1545	Cohort study	B
**Rubinstein2013** [29]	7293	23,195	Cross sectional study	B
**Sheffield2012** [30]	8690	76,153	Cohort study	B
**Steinhoff2012** [31]	161	166	Randomized trial	B
**Steinhoff2017** [32]	1847	1846	Randomized trial	B
**Zerbo2017** [33]	64,748	81,119	Cohort study	B
**Total no of participants (n)**	181,479	498,513		

As listed in Table 3, the average percentage of pregnant women who had gestational diabetes (GDM) ranged between 1.06 and 16.2%, those with hypertension ranged from 0.42 to 51.9%, those with multiple pregnancies ranged from 1.00 to 5.80%.

**Table 3:** Baseline features reported by the participants

Studies	GDM (%) Vac/UV	HBP (%) Vac/UV	MP (%) Vac/UV	FA suppl (%) Vac/UV
**Baum2015** [15]				
**Beau2014** [16]	3.10/3.80	0.90/1.20	2.10/2.50	27.4/18.5
**Chambers2013** [17]		8.90/3.90		69.0/72.3
**Chambers 2016**[18]		8.10/5.50		72.1/63.8
**Cleary2014** [19]	–	–	–	–
**Fabiani2015** [20]	–	–	2.00/1.30	–
**Fell2012** [21]		48.1/51.9	–	–
**Kallen2012** [22]	1.06/1.84	0.42/0.74	–	–
**Legge2014** [23]	4.80/4.98	1.02/1.11	–	–
**Lin2012** [24]	–	–	–	–
**Maas2015** [25]	–	–	–	–
**Olsen2016** [26]	–	–	–	–
**Pasternak2012** [27]	–	–	–	–
**Richards2013** [28]	16.2/15.6	14.4/14.9	5.30/5.80	–
**Rubinstein2013** [29]	–	4.10/3.60	–	–
**Sheffield2012** [30]	12.0/6.00	9.00/9.00	2.00/1.00	–
**Steinhoff2012** [31]	–	–	–	–
**Steinhoff2017** [32]	–	–	–	–
**Zerbo2017** [33]	–	2.10/1.87	–	–

Abbreviations: *GDM* Gestational diabetes, *HBP* High blood pressure, *MP* Multiple pregnancy, *FA suppl* Folic acid supplement, *Vac* Vaccination, *UV* Unvaccinated

### Main results

**Fig. 2:**
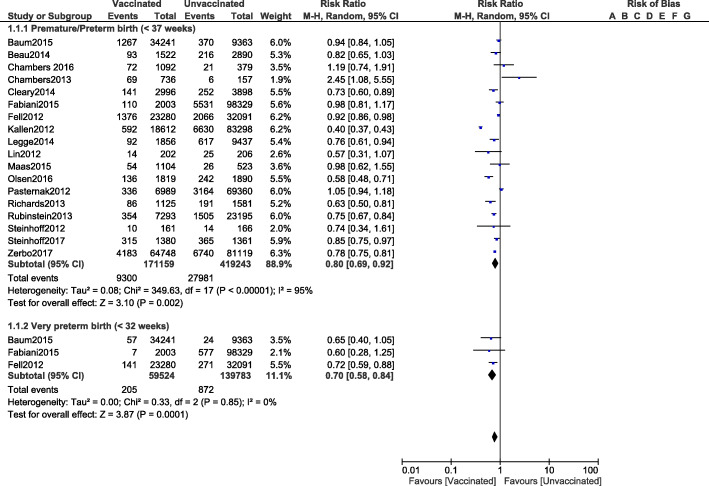
Comparing the adverse fetal outcomes in pregnant women who were vaccinated versus who were not vaccinated for Influenza A (Part I)

Based on this analysis, premature/preterm birth (< 37 weeks) was significantly reduced in pregnant women who were vaccinated for influenza A (RR: 0.80, 95% CI: 0.69–0.92; *P* = 0.002) as compared to those women who were not vaccinated as shown in Fig. 2. Similarly, influenza A vaccination also decreased the risk for very preterm birth (< 32 weeks) (RR: 0.70, 95% CI: 0.58–0.84; *P* = 0.0001) as shown in Fig. 2.

**Fig. 3:**
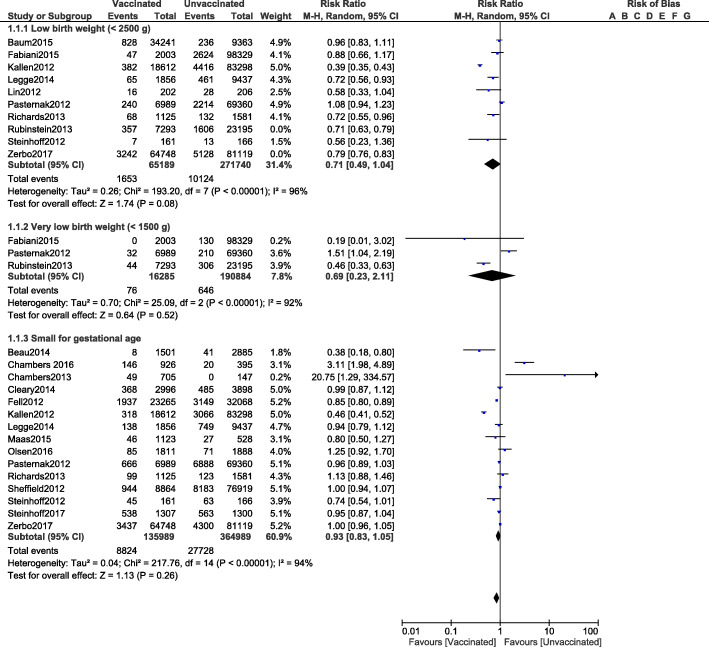
Comparing the adverse fetal outcomes in pregnant women who were vaccinated versus who were not vaccinated for Influenza A (Part II)

The risks for infants with low birth weight (RR: 0.71, 95% CI: 0.49–1.04; *P* = 0.08), very low birth weight (RR: 0.69, 95% CI: 0.23–2.11; *P* = 0.52) and infants small for gestational age (RR: 0.93, 95% CI: 0.83–1.05; *P* = 0.26) were not increased in the vaccination group as shown in Fig. 3.

**Fig. 4:**
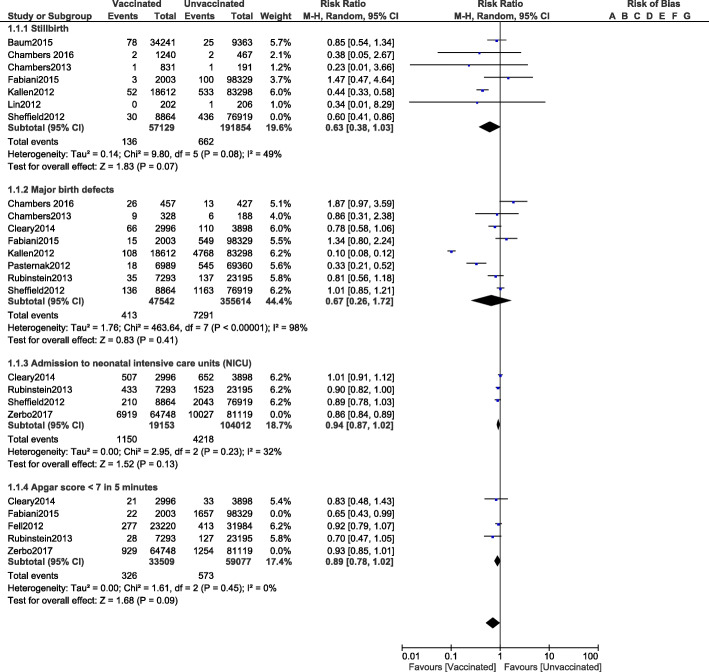
Comparing the adverse fetal outcomes in pregnant women who were vaccinated versus who were not vaccinated for Influenza A (Part III)

Influenza A vaccination was not associated with increased risks of stillbirth (RR: 0.63, 95% CI: 0.38–1.03; *P* = 0.07), birth defects (RR: 0.67, 95% CI: 0.26–1.72; *P* = 0.41), admission to the neonatal intensive care unit (RR: 0.94, 95% CI: 0.87–1.02; *P* = 0.13) or an Apgar score < 7 in 5 min (RR: 0.89, 95% CI: 0.78–1.02; *P* = 0.09) as shown in Fig. 4.

**Fig. 5:**
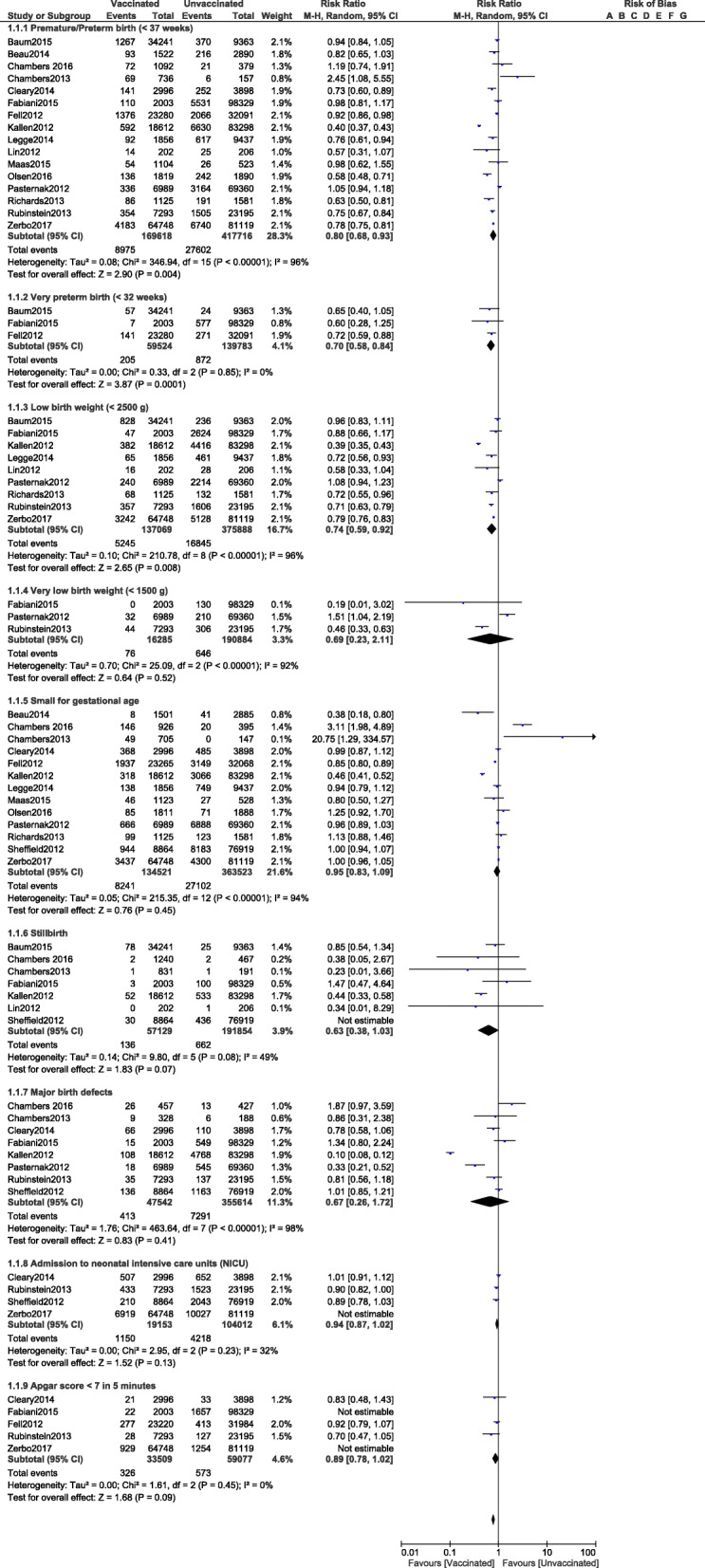
Comparing the adverse fetal outcomes in pregnant women who were vaccinated versus who were not vaccinated for Influenza A using data only from observational studies

When the two randomized trials were excluded, and an analysis was carried out only with the observational cohorts, preterm birth was still significantly lower among women who were vaccinated for influenza A (RR: 0.80, 95% CI: 0.68–0.93; *P* = 0.004) as shown in Fig. 5. Infants with low birth weight were also significantly reduced (RR: 0.74, 95% CI: 0.59–0.92; *P* = 0.008). Infants who were small for gestational age (RR: 0.95, 95% CI: 0.83–1.09; *P* = 0.45), stillbirth (RR: 0.63, 95% CI: 0.38–1.03; *P* = 0.07), major birth defects (RR: 0.67, 95% CI: 0.26–1.72; *P* = 0.41), admission to NICU (RR: 0.94, 95% CI: 0.87–1.02; *P* = 0.13) and Apgar score < 7 in 5 min (RR: 0.89, 95% CI: 0.78–1.02; *P* = 0.09) were also not increased as shown in Fig. 5.

A summarized version of the result based on the safety of influenza A vaccination during pregnancy has been provided in Table 4.

**Table 4:** Results of the analysis

Endpoints which were assessed	RR with 95% CI	***P*** values
**Premature/Preterm birth (< 37 weeks)**	0.80 [0.69–0.92]	0.002
**Very preterm birth (< 32 weeks)**	0.70 [0.58–0.84]	0.0001
**Low birth weight (< 2500 g)**	0.71 [0.49–1.04]	0.08
**Very low birth weight (< 1500 g)**	0.69 [0.23–2.11]	0.52
**Small for gestational age**	0.93 [0.83–1.05]	0.26
**Stillbirth**	0.63 [0.38–1.03]	0.07
**Major birth defects**	0.67 [0.26–1.72]	0.41
**Admission to NICU**	0.94 [0.87–1.02]	0.13
**Apgar score < 7 in 5 min**	0.89 [0.78–1.02]	0.09

Abbreviations: *RR* Risk ratios, *CI* Confidence intervals, *NICU* Neonatal intensive care unit

**Fig. 6:**
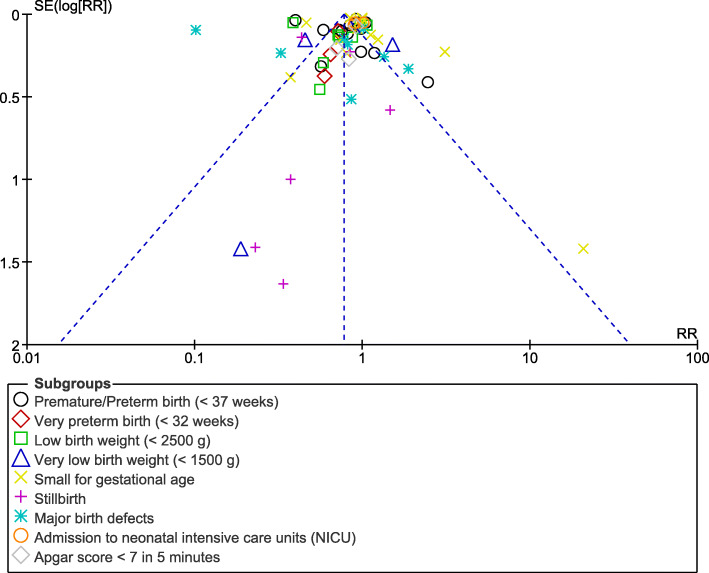
Funnel plot showing publication bias

Consistent results were obtained throughout based on a sensitivity analysis. Publication bias was represented by the funnel plot (Fig. 6).

## Discussion

Our results were evidence to prove that influenza A vaccination in pregnancy was associated with a significantly lower risk of preterm birth and without increasing the risk of other adverse fetal outcomes including stillbirth, congenital malformations, poor Apgar score and admission to NICU showing a benefit of this vaccine in pregnancy.

Similarly, studies based in the United States of America and Europe demonstrated that maternal influenza vaccine is associated with a significantly high level of anti-influenza antibody thus showing a benefit for the mother as well as for the infant [36].

A recent systematic review and meta-analysis of observational studies found that influenza when manifested during pregnancy resulted in an increased risk of hospital admission compared to the non-pregnant controls [37]. This indicates that influenza vaccine during pregnancy is vital to reduce hospital admission and this particular vital piece of information should be communicated to the mass.

A conference report of the World Health Organization (WHO) technical consultation on the effect of maternal influenza and influenza vaccination on the development of the fetus: Montreal, Canada, September 30 – October 1, 2015 has been described [38].

In a large retrospective cohort including 74, 292 participants retrieved from seven Vaccine Safety Data-link sites which is a collaborative effort between the Immunization Safety Office of the centers for Disease Control and Prevention involving approximately 3% of the population of the United States, the authors demonstrated no association of influenza vaccine with increased adverse obstetrical events [39]. Another study also showed no associated risk of influenza vaccine on pregnancy outcomes [40].

Another cohort study from Denmark comprising of 54, 585 pregnancies whereby 7062 women were vaccinated against influenza, no evidence of fetal death was observed with this vaccine given during pregnancy [41]. All those studies from different regions across the world are evidence to suggest the benefits of influenza A vaccine during pregnancy. This particular health communication should be spread across the concerned population of pregnant and non-pregnant women, as well as to their families. This public health issue is vital, for a healthier generation.

For a long time, people were affected by the sensitivity of pregnancy, and the particularity of vaccine and the complexity of influenza virus, faced many difficulties and challenges for a long time in scientific popularization, health education, risk communication and vaccine promotion of influenza A vaccination during pregnancy. Examples were: “does influenza A vaccination during pregnancy have side effects?,” “Will health communication on influenza A vaccination during pregnancy be rejected by those conservative women?.” In view of these issues, we must carefully and detailly operate our researches, and design communication strategies for influenza A vaccination in pregnancy.

Theory is the guide to practice, and this is of no exception for health communication based on influenza A vaccination in pregnancy. From the perspective of this issue, directly relevant theories on health communication were mainly presented as: Perception of Risks theory and Social Determinants of Health theory. Risk perception refers to individual’s subjective cognition and judgment on any type of objective risks outside, yet risk perception on the health particularly refers to those cognitions and judgment of public on various factors, activities and common diseases that affect physical and mental health [42]. “Risk perception” plays an indispensable role in health and risk communication, which can help us investigate what concerns exist among the public and understand people’s related health behaviors, such as searching, selecting, and sharing health information. Because, a fact that cannot be ignored is that when people perceive potential health risks, they are often prone to falling into negative emotions, such as anxiety or even fear, and driven by the self-protection, they will actively seek information and solutions to change the current uneasy state. Wu Hairong and Shen Ying [43] studied and proved that community residents were allowed to understand the hazards of rabies and the risks of bites by dogs and cats, could improve their awareness of prevention, attitude and behavior of rabies (KAP); Brewer NT [44] also demonstrated that there exists a positive correlation between risk perception and health behaviors, that is, individuals ‘awareness of health risks can increase their concerns about health risks and their compliance with actions. As to this phenomenon, the Protection Motivation Theory (PMT) also gives a similar explanation. It is in the above sense that we can say without hesitation: Risk perception constitutes an effective predictor of decision-making and intervention for health. In addition, the Health Social Factor Theory (SDOH) emphasizes that people’s health risks and quality of life are affected by people’s living resources, educational opportunities, medical and health services, community environment, and social norms, to some extent. WHO [45] states that if we want to improve people’s health worldwide, we must do three basic tasks: educational intervention, social protection intervention and urban development intervention. Yet, education intervention is the so-called direct health communication intervention.

Based on the above two theories, we focus on advocating the following skills and methods for the communication of influenza A vaccination during pregnancy, to accomplish the special social issue into a larger spread in pregnant women and society. In general, for the sake of reducing doubts and eliminating traditional misunderstanding from the pregnant women and the public, we must adhere to the principle of “active behavior, scientific guidance”-based on facts and science, to guide them to correctly understand and judge the safety of vaccines. Specifically, the health communication and education on this topic can be strengthened and improved from such aspects, as follows:

(1) To Enter correct and scientific health information for preventing fatalism and nihilism in heath communication. Fatalism and nihilism are often synonymous with meaninglessness, hopelessness, and inaction. Related researches in the field of health communication, also shows that thus beliefs constitute an invisible wall and a major barrier for our carrying out medical care, which affects disease prevention, early detection and treatment [46, 47]. Influenced by fatalism, people believe that health problems are not under control of human beings and are “destined” to be unsolvable. Fatalism also exists on the issue of influenza A vaccination in pregnancy. Many women, for example, still believe that “there is no way to prevent the occurrence and spread of influenza virus”, “there is no way to reduce the possibility of infection by influenza virus”, “even if influenza A vaccination in pregnancy is safe, it will not escape from its side effects.” According to fatalism, it makes no sense to operate health communication and educational intervention, which is diametrically opposed to risk perception theory. The latter insists that public, once realizing the health risks, will tend to actively seek information to avoid risks. Also, Practice and a large number of studies have confirmed that a significant positive correlation exist between people’s health communication/interventions for risks and their behaviors for health. Therefore, we must make a difference in the safety of influenza A vaccination during pregnancy, through filtering and visual presentation to input correct and scientific health information towards pregnant women and the public, and prevent their negative attitudes to health behaviors. In addition to considering those actual needs in society and adopting those communication methods popular with the public, relevant news production and its spread must be continuous for the safety of influenza A vaccination during pregnancy, rather than in an intermittent state. Only by a long-term guidance can people’s traditional perception for the event be effectively changed.

(2) Public institutions for health education should make full use of authoritative media, especially social media, to promote the penetration and reach of health information. In the era of mass communication, the media, like traditional schools and churches, undertake important responsibilities in social enlightenment and cultural education. Especially with the advent of social media and mediated society, the way people understand the world and the interactions between them have gradually become socialized and stratified. Social media, due to its efficient interaction, diverse structure and advantages of crossing spaces, has turned into the mainstream media in our society [48]. This communication phenomenon also exists in the realm of health communication. An American survey in 2017 showed that 74% of Internet users surfed on social media; 80% of them searched for health information; 30% of adults used social media to share the health information with other patients [49]. Given the media and social platforms have been an important channel for health communication, in which people search for, write, evaluate and share health information, detailed and authoritative facts/truths about the safety of influenza vaccines, should be provided to pregnant women through the media and especially social networks by public medical institutions for health and popular science, and a deeply interaction with the audience, be operated through new media’s flat and multi-polar characteristics to answer questions, enhance mutual trust/ the penetration of heath information, and reduce false information. Furthermore, about the influenza A vaccination during pregnancy, relevant organizations can also use social media to create topic groups (an effective way to gather target audiences), and bring humanistic care to the communication, which contributes to a comprehensive interaction between doctors and audience, and comforts pregnant women with the support and encouragement from those who have been vaccinated.

(3) To cultivate professional “opinion leaders” for health communication and value realistic community communication, SDOH theory believes that individual’s cognitions and attentions for health risks are also affected by social factors. Hence, a type of reasonable health communication and interventions must be implemented, for the improvement of public health. Obviously, the communication and persuasion for influenza A vaccination during pregnancy, an indispensable part of public health, are not only vitally interrelated with the intrinsic content and quality of information, but depend on other factors, such as communicators’ credibility, and the community environment/culture in which the audience live. Especially in those cultural systems that value traditions and collectivism, SDOH theory has much more considerable vitality in explaining public KAP model for health information. In light of this, through cultivating professional opinion leaders for health communication and interventions may we be able to make it widely known towards women and society that influenza A vaccination is securely-guaranteed. Genuinely speaking, slightly more materials, money and time may be consumed in “fostering opinion leaders” (such as well-known scholars and scientist in the medical field), and “drawing support from traditional community communication” (such as doctor’s visits and on-site education in public occasions), compared with the communication and intervention by social media. However, these methods above all will absolutely enhance mutual trust and effective communication for health information in a way that more lives up to people’s psychology.

In this study, the authors focused on the effect of influenza A/H1N1pdm09 vaccine on fetal outcomes, although a quadrivalent influenza vaccine has also usually been used in the general influenza season. To support monovalent vaccination as in this current study, another research article [50] based on the immunogenicity and efficacy of the monovalent, trivalent and quadrivalent intranasal live attenuated influenza vaccines containing different pdmH1N1 strains showed viral titres in the nose to have significantly been reduced in animals who were vaccinated with monovalent vaccines compared to those who were vaccinated with trivalent and quadrivalent vaccines of both the strains and it was only the monovalent vaccines containing the A/Cal strain that significantly reduced the viral load. In conclusion, the authors also stated that the monovalent vaccines appeared to be superior and provided complete protection from infections during a pandemic. Monovalent vaccines has other advantages. A meta-analysis even showed that pregnant women who received monovalent vaccines for H1N1 were less likely to deliver low birth weight babies [51]. Another published article showing variable influenza vaccine effectiveness by subtype also showed monovalent vaccine to be more effective [52]. The study showed that vaccine efficiency against influenza type B and H1N1pdm09 was greater than 50% among all age group. Even though this current analysis was based on monovalent influenza vaccine, a recent randomized, observer-blind trial based on the immunogenicity and safety of the quadrivalent inactivated influenza vaccine in pregnant women showed the latter to be equally safe [53]. No vaccine related adverse pregnancy outcomes or congenital malformations were reported. However, since it was the first study to evaluate the efficacy of quadrivalent influenza vaccine in pregnant women, with a limited number of participants and since the study only included 2nd and 3rd trimester pregnant women, it would be recommended to better await further studies to confirm the safety of the quadrivalent influenza vaccine in pregnant women.

At last, one of the limitations of this meta-analysis was a high level of heterogeneity when assessing several subgroups. However, it should be noted that data from several studies including randomized trials, cohort studies and cross sectional studies were included and the introduction of bias was obvious as stated in a vaccine-related article [54]. Also, even if heterogeneity was very high in most of the cases, no graphical representation [55] was included to show which articles were more influential in heterogeneity because we already carried out a leave one out analysis. In addition, the time period when the vaccine was given was not taken into consideration whether it was given during the first, second or third trimester believing that it was at least given during pregnancy. Furthermore, the presence of covariates, selection bias and other types of bias might have affected the results. Also, several other baseline features were not reported in the original studies, and hence, we were unable to include some more details about these baseline characteristics of the pregnant women in this analysis.

## Conclusion

Influenza vaccine is completely safe in pregnancy. It significantly lowers premature birth and is not associated with any serious adverse neonatal outcome. Hence, this important piece of information should be communicated and conveyed to all pregnant women, for a safer and healthier pregnancy. At last, this public health issue should further be addressed to the population through media and other communication means in order to improve the rate of influenza A vaccines in pregnant women for a healthier and more productive population.

## Data Availability

All data and materials used in this research are freely available in electronic databases (MEDLINE, EMBASE, Cochrane database, Google scholar). References have been provided.

## Abbreviations

SHDPP: Stanford Heart Disease Prevention Program
CDC: Centers for Disease Control and Prevention
H1N1: Influenza A
SAGE: Strategic Advisory Group of Experts on Immunization
RR: Risk ratio
CI: Confidence interval
WHO: World Health Organization
